# Temperature Compensation of Wind Tunnel Balance Signal Detection System Based on IGWO-ELM

**DOI:** 10.3390/s23167224

**Published:** 2023-08-17

**Authors:** Xiang Dong, Hu Xu, Huibin Cao, Tao Cui, Yuxiang Sun

**Affiliations:** 1 School of Electrical Engineering and Automation, Anhui University, Hefei 230601, China; xdong@ahu.edu.cn (X.D.); z21301065@stu.ahu.edu.cn (H.X.); 2 Hefei Institutes of Physical Science, Chinese Academy of Sciences, Hefei 230031, China; 3 Department of Science Island, University of Science and Technology of China, Hefei 230026, China; 4 Chengdu Science and Technology Development Center of CAEP, Chengdu 610200, China; cuit0013@yinhe596.cn

**Keywords:** wind tunnel balance, signal detection system, temperature compensation, gray wolf optimization algorithm, extreme learning machine

## Abstract

The wind tunnel balance signal detection system is widely employed in aerospace applications for the accurate and automated measurement of aerodynamic forces and moments. However, measurement errors arise under different environmental temperature. This paper addresses the issue of measurement accuracy under different temperature conditions by proposing a temperature compensation method based on an improved gray wolf optimization (IGWO) algorithm and optimized extreme learning machine (ELM). The IGWO algorithm is enhanced by improving the initial population position, convergence factor, and iteration weights of the gray wolf optimization algorithm. Subsequently, the IGWO algorithm is employed to determine the optimal network parameters for the ELM. The calibration decoupling experiment and high-low temperature experiment are designed and carried out. On this basis, ELM, GWO-ELM, PSO-ELM, GWO-RBFNN and IGWO-ELM are used for temperature compensation experiments. The experimental results show that IGWO-ELM has a good temperature compensation effect, reducing the measurement error from 20${\%}_{FS}$ to within 0.04${\%}_{FS}$. Consequently, the accuracy and stability of the wind tunnel balance signal detection system under different temperature environments are enhanced.

## 1. Introduction

The wind tunnel balance is an experimental device widely utilized in the study of aerodynamics [1]. It comprises a multi-component force measurement sensor designed to measure the aerodynamic forces and moments acting on various models, including spacecraft and automobiles. The balance incorporates multiple full-bridge circuits composed of strain gauges, enabling the sensing of changes in aerodynamic forces and moments in all dimensions [2]. During testing, aerodynamic loads can be determined by supplying excitation to the six-axis balance circuit using a high-precision power supply and capturing the response signal of the balance through the acquisition of a signal detection system [3]. In practical applications of the six-axis balance signal detection system, different working environments can have an impact on the detection system, particularly the influence of ambient temperature. When operating under extreme conditions, the wide range of ambient temperature can induce significant drift in the system’s characteristics. The characteristic drifts will cause bias between measured values and real values, thereby affecting measurement accuracy [4]. Therefore, compensating for the temperature effect on the balance signal detection system is crucial, as the characteristic drift caused by temperature profoundly affects the accuracy of balance testing.

The effects of temperature on the balance signal detection system can be mainly attributed to two components: the elastic bodies and the signal conditioning circuit. The elastic bodies component comprises the elastic body and the strain gauges affixed to its surface. The temperature generated error is primarily caused by the thermal effects on the strain gauges and the disparate expansion coefficients between the strain gauges and elastic bodies. These factors play a crucial role in inducing temperature generated error in the elastic bodies. The signal conditioning circuit consists of various modules, including the excitation source, zero adjustment, anti-electromagnetic filter, amplifier circuit, low-pass filter, and reference source circuit. The entire signal conditioning circuit is susceptible to temperature effects, resulting in changes in the temperature coefficient and voltage drift of the amplifier. These temperature-induced variations, in turn, cause changes in the output value of the circuit. Additionally, apart from resistors and amplifiers, the excitation source, reference source, and capacitors within the conditioning circuit also exhibit distinct characteristics with temperature changes. Collectively, these factors contribute to the characteristic drift phenomenon observed in the balance signal detection system [5].

The compensation of the balance signal detection system for temperature can be achieved through hardware compensation or software compensation methods. Hardware compensation involves designing the circuit to eliminate temperature coefficients [6]. For instance, Chu et al. [7] adopted double thermocouples to measure the airflow temperature and proposed a time domain compensation method to compensate for the airflow temperature measurement results. Wu et al. [8] presented a method that utilizes pulsed light received by a Faraday rotating mirror to differentiate between the temperature and current. Lee et al. [9] involved a fluorescence quenching-based gas sensor and a resistive temperature detector (RTD) with an integrated thermometer for in situ temperature monitoring and compensation. However, hardware compensation is more costly, subject to material-processing constraints, and introduces new uncertainties to the system, limiting its compensation accuracy. On the other hand, software compensation involves constructing a compensation model to obtain the compensated output voltage value using the measured voltage and real-time temperature values. Software compensation is more cost-effective, flexible, and accurate than hardware compensation, making it more suitable for practical applications. Numerous software compensation methods have been proposed by researchers, such as least squares method (LSM), wavelet transform (WT) [10], and various artificial neural networks (ANNs) [11,12,13,14]. The LSM is more suitable for linear problems, and the error caused by temperature is usually non-linear. And WT requires a large number of parameters for model building, making it complex and challenging to implement. In recent years, artificial neural networks, such as backpropagation (BP) and least squares support vector machine (LSSVM) [15,16] models, have gained popularity for temperature compensation. However, these models tend to be more complex, prone to overfitting, and require a significant number of parameters. In contrast, the ELM model [17] is simple yet possesses strong generalization abilities, making it more suitable for addressing overfitting issues. For example, Li et al. [18] proposed an ELM-based temperature compensation method for piezoresistive differential pressure sensors. Another study by Long et al. introduced a temperature-compensated ELM model for low coupling and temperature compensation [19].

Although ELM demonstrates efficient and accurate performance in regression prediction, its random generation of input layer weights and implicit layer thresholds can lead to network instability. To address this issue, optimization algorithms are utilized to optimize the weights and thresholds of ELM, thereby reducing prediction error values, improving network performance, and enhancing the ELM prediction accuracy. In recent years, the field of metaheuristic algorithms has seen rapid development, with examples including particle swarm optimization (PSO) [20], the sparrow search algorithm (SSA) [21], the whale optimization algorithm (WOA) [22], the fireworks algorithm (FWA) [23], and the gray wolf optimization (GWO) [24,25], among others. GWO offers a faster optimization process compared to other algorithms, as it first derives the solutions and then ranks and compares them to obtain the best solution. However, GWO has drawbacks, such as difficulty in handling a large number of variables and escaping local optima when solving large-scale problems [26]. In a previous study, Xie et al. proposed a three-stage update function for inertia weights and a dynamic update method for learning rate to enhance GWO performance by avoiding local optima [27]. Jin et al. proposed optimal Latin hypercube sampling initialization, nonlinear convergence factor, and dynamic weights for IGWO to enhance its accuracy in optimizing support vector regression (SVR) parameters [28]. Therefore, this paper aims to improve the gray wolf algorithm by enhancing three aspects: initial population location, convergence factor, and iteration weights.

Based on the aforementioned discussions, this paper employs the IGWO to optimize the ELM neural network, aiming to obtain the optimal weights and thresholds for the network. Building upon this, we propose the IGWO-ELM for the temperature compensation method. In this study, we focus on the calibration decoupling experiments, high- and low-temperature experiments, and temperature compensation experiments using a six-axis force/torque sensor and a balance signal detection system board applied to the space robotic arm as our research object. The experimental results demonstrate that IGWO-ELM exhibits a remarkable temperature compensation effect and demonstrates excellent stability.

The main contributions are as follows:

- This paper proposes three improvements to enhance the performance of the gray wolf algorithm:
  1. Improving the initialized population by employing circle chaotic mapping to enhance the diversity of the initial population, thus promoting exploration in the search space;
  2. Enhancing the convergence factor by utilizing a nonlinear function, which enhances the global search capability in the early stages of the algorithm and improves the convergence speed in the later stages;
  3. Accelerating the convergence speed of the algorithm towards the optimal solution by enhancing the dynamic weighting strategy.

- Presents a temperature compensation method for the wind tunnel balance signal detection system based on the IGWO algorithm and the ELM model. The proposed method aims to accurately predict and compensate for the errors induced by temperature in the system. By utilizing the IGWO algorithm and the ELM model, the temperature-related errors can be effectively mitigated, leading to improved measurement accuracy and reliability of the wind tunnel balance signal detection system.
- The calibration decoupling experiment and high–low temperature experiment are designed and carried out. On this basis, ELM, GWO-ELM, PSO-ELM, GWO-RBFNN and IGWO-ELM are used for temperature compensation experiments. The experimental results show that IGWO-ELM has a good temperature compensation effect, and the measurement error is reduced from 20${\%}_{FS}$ to less than 0.04${\%}_{FS}$.

## 2. Basic Algorithm

### 2.1. Extreme Learning Machine $\left(ELM\right)$

**Figure 1:**
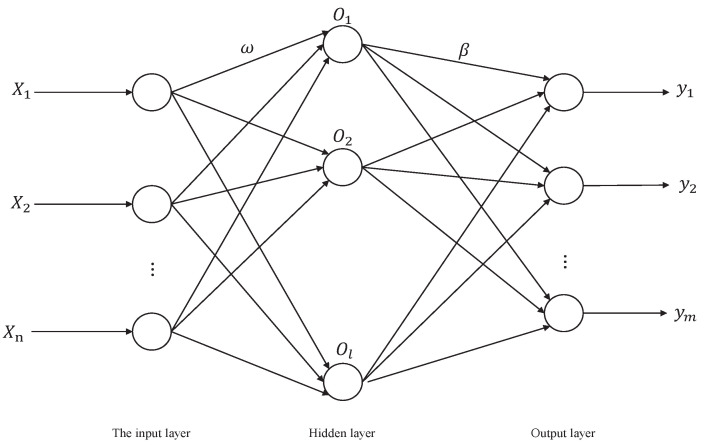
ELM network structure.

ELM is a feed-forward neural network model for machine learning that offers several advantages over other models, such as BPNN and RBFNN. ELM models [29] do not require the adjustment of structural parameters during training, making them simple and efficient. In ELM, the connection weights between the input layer and the hidden layer, as well as the thresholds of the hidden layer neurons, are randomly generated and do not require adjustments during training. The only requirement is to set the number of neurons in the hidden layer to obtain the optimal solution. The network structure is illustrated in Figure 1.

Suppose there are N training samples $\left(X_{i},Y_{i}\right)$, where 1≤i≤N, $X_{i}={\left[X_{i1},X_{i2},\cdots ,X_{in}\right]}^{T}$$\in R^{n}$ is the input vector of the *i*th sample, and $Y_{i}={\left[y_{i1},y_{i2},\cdots ,y_{im}\right]}^{T}\in R^{m}$ is the output vector of the *i*th sample, then the ELM network can be represented as

(1) $$\sum_{l}^{f}\beta_{f}g\left(W_{f}X_{i}+b_{f}\right)=T_{i}$$

where $g\left(x\right)$ is the activation function; $W_{f}={\left[w_{f1},w_{f2},\cdots ,w_{fn}\right]}^{T}$ is the input weight; $b_{f}$ is the threshold of the *f*-th hidden layer neuron; $\beta_{f}=\left[\beta_{f1},\beta_{f2},\cdots ,\beta_{fm}\right]$ is the output weight; and $T_{i}={\left[t_{i1},t_{i2},\cdots ,t_{im}\right]}^{T}$ is the output vector of the *i*th sample.

The learning goal of ELM is to minimize the output error of the network as much as possible, expressed as

(2) $$\sum_{i=1}^{N}\left|\left|T_{i}-Y_{i}\right|\right|=0$$

That is, there exists a suitable set of *W*, *b* and β such that

(3) Hβ=Y

Among them, $H=\left[\begin{array}{ccc} g\left(W_{1}X_{1}+b_{1}\right) & \cdots & g\left(W_{l}X_{1}+b_{l}\right)\\ \vdots & \ddots & \vdots \\ g\left(W_{1}X_{N}+b_{1}\right) & \cdots & g\left(W_{l}X_{N}+b_{l}\right) \end{array}\right]$ represents the implied layer output matrix of the network; $\beta ={\left[\beta_{1}^{T},\beta_{2}^{T},\cdots ,\beta_{l}^{T}\right]}_{l\times m}^{T}$; and $Y={\left[Y_{1}^{T},Y_{2}^{T},\cdots ,T_{N}^{T}\right]}_{N\times m}^{T}$. The output weights β can be obtained by solving the least squares solution of the following system of equations:

(4) $$\min_{\beta }=\left|\left|H\beta -Y\right|\right|$$

The solution is

(5) $$\hat{\beta }=H^{+}Y$$

where $H^{+}$ is the generalized inverse matrix of the output matrix *H* of the hidden layer.

### 2.2. Gray Wolf Optimization Algorithm $\left(GWO\right)$

**Figure 2:**
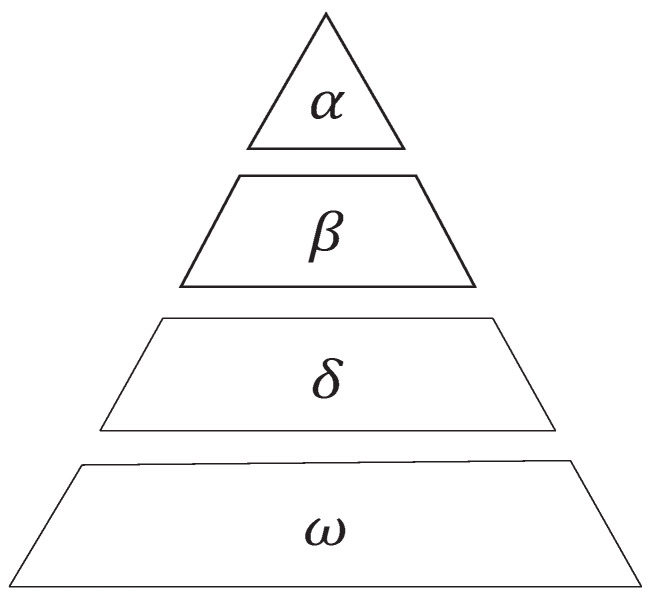
Gray wolf hierarchy.

The gray wolf optimization algorithm is a novel meta-heuristic algorithm that draws inspiration from the internal system and hunting patterns of gray wolves in nature. It aims to find optimal solutions by emulating the hunting behavior of gray wolf packs. In the natural environment, gray wolves occupy a higher position in the food chain and exhibit a preference for group living. Within the gray wolf community, a strict hierarchical structure is maintained, as depicted in Figure 2.

**Figure 3:**
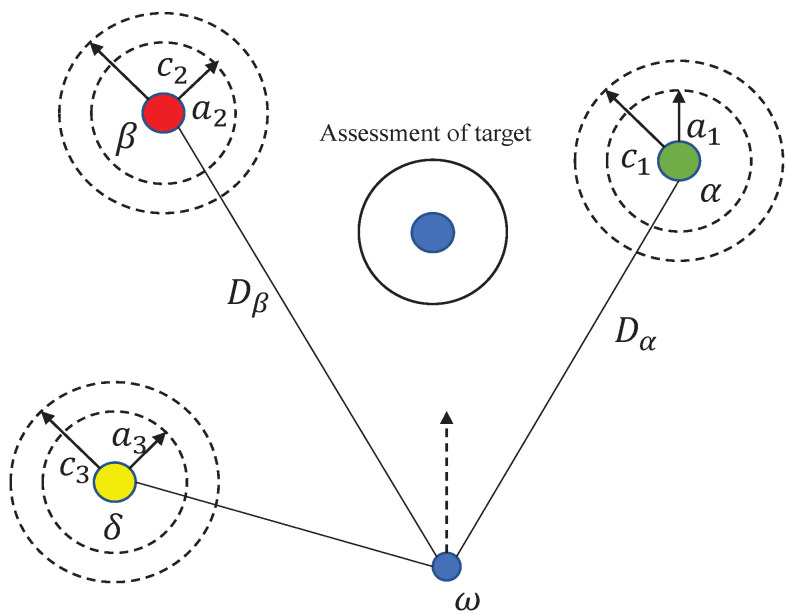
Schematic diagram of the gray wolf algorithm for finding advantages.

The GWO classifies wolf packs into four categories. The first category consists of α wolves, who hold the dominant position and are primarily responsible for decision making regarding hunting and food distribution. The second category comprises β wolves, who strictly follow the leadership of α wolves and serve as mediators for transmitting information. In the absence of an α wolf, β wolves can assume control over all other wolves, except those in the first category. The third category encompasses δ wolves, who only dominate ω wolves and are mainly responsible for fundamental tasks within the pack, such as standing guard and scouting. Lastly, the fourth category comprises ω wolves, who occupy the lowest position in the pack’s hierarchy and are tasked with executing orders from higher-ranking wolves and maintaining pack harmony. Gray wolves show collective predation, characterized by encirclement, hunting, and attack strategies. In the GWO algorithm, the first three optimal solutions correspond to the positions of α, β, and δ wolves, while the remaining solutions represent positions of ω wolves. In each iteration, the optimal position is estimated using the first three optimal solutions. The gray wolves are then guided to randomly update their positions around these optimal solutions, gradually converging towards the global optima. Figure 3 illustrates the schematic diagram of the optimal search process.

1. Surrounding behavior
  Throughout the encirclement process, the separation distance, denoted as *D*, between the wolf pack and the prey, is captured by the formulation outlined in Equation (6). To adapt their positions dynamically, the wolf pack undergoes position updates contingent upon the aforementioned distance as delineated in Equation (7). By manipulating the coefficient vectors *A* and *C*, it becomes possible to guide the wolves towards the prey from varied vantage points. The determination of these coefficients is facilitated through the deployment of Equations (8) and (9):
  (6) $$D=\left|CX_{p}\left(t\right)-X\left(t\right)\right|$$
  (7) $$X\left(t+1\right)=X_{p}\left(t\right)-AD$$
  (8) $$A=2ar_{1}-a$$
  (9) $$C=2r_{2}$$
  where *t* is number of current iterations; $X_{p}$ is the position vector of the prey; and *D* is the distance. *a* is the convergence factor, slowly decreasing from 2 to 0, and $r_{1},r_{2}$ are random numbers in $\left[0,1\right]$.
2. Hunting behavior
  Once the wolf pack has successfully encircled the prey, the α wolf, β wolf, and δ wolf position themselves closest to the prey. Under the leadership of these wolves, the entire pack advances towards the prey. The positional hunting behavior of the ω wolf can be described by the following mathematical model:
  (10) $$D_{\alpha }=C_{1}X_{\alpha }\left(t\right)-X\left(t\right)$$
  (11) $$D_{\beta }=C_{2}X_{\beta }\left(t\right)-X\left(t\right)$$
  (12) $$X_{1}\left(t+1\right)=X_{\alpha }\left(t\right)-A_{1}D_{\alpha }$$
  (13) $$X_{2}\left(t+1\right)=X_{\beta }\left(t\right)-A_{2}D_{\beta }$$
  (14) $$X_{3}\left(t+1\right)=X_{\delta }\left(t\right)-A_{3}D_{\delta }$$
  (15) $$X\left(t+1\right)=\frac{X_{1}\left(t+1\right)+X_{2}\left(t+1\right)+X_{3}\left(t+1\right)}{3}$$
3. Attacking the prey
  The objective of this phase is to capture the prey, which corresponds to obtaining the optimal solution. In the GWO algorithm, the process of approximating the prey is simulated by gradually decreasing the value of parameter *a*. As *a* decreases, the elements of vector *A* are constrained to the interval [−a,a]. When $\left|A\right|$ < 1, the wolves can attack the prey; conversely, when $\left|A\right|$ > 1, the wolves disperse in various directions, leading to a loss of the optimal position. This behavior highlights the tendency of the GWO algorithm to become trapped in local optima.

### 2.3. Improved Gray Wolf Optimization Algorithm $\left(IGWO\right)$

#### 2.3.1. Optimize Initial Population Location

**Figure 4:**
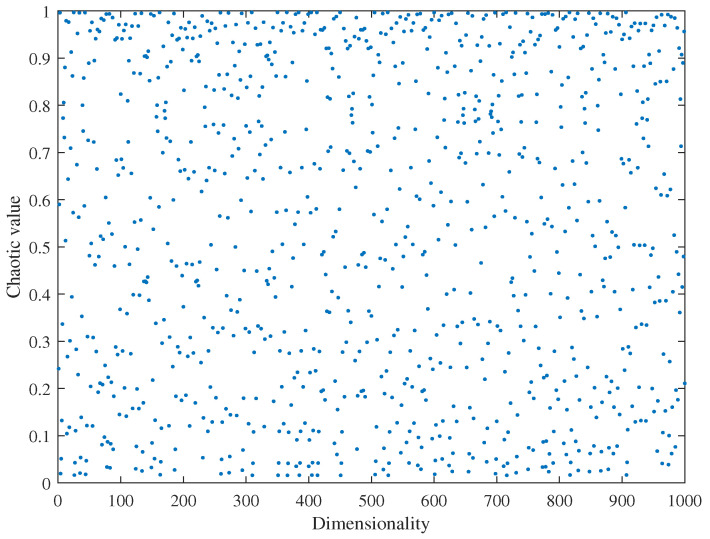
Circle chaotic mapping scatter plot.

Chaotic motion possesses significant advantages, such as regularity, randomness, and ergodicity. To increase population diversity and prevent the algorithm from getting trapped in local optima during the optimization process, the concept of chaotic mapping is incorporated into the GWO algorithm. Various methods can be used for chaotic mapping, including logistic mapping, tent mapping, and circle mapping [30]. However, it has been shown that the distribution of logistic mapping is nonuniform and biased towards the extremes, which adversely affects the speed and accuracy of finding the optimal solution [31]. Tent mapping [32] exhibits a more uniform distribution, but it is prone to convergence towards stationary points. In comparison, circle mapping demonstrates a more stable distribution with uniform values across the range as depicted in Figure 4. Therefore, in this paper, circle chaotic mapping is employed to initialize the population of the gray wolf algorithm.The expression for circle chaotic mapping is as follows:

(16) $$X_{i+1}=mod\left(X_{i}+0.2-\left(0.5/2\pi \right)\sin\left(2\pi X_{i}\right),1\right)$$

where $X_{i}$ is the *i*-th dimensional random value.

#### 2.3.2. Optimized Convergence Factor

The GWO algorithm’s ability to balance exploration and exploitation is influenced by the parameter *A*, and the convergence factor *a* determines the value of *A*. In each iteration, the convergence factor *a* is linearly reduced from 2 to 0. However, the GWO algorithm does not solve problems in a linear optimization process. Therefore, the linear decrease in *a* does not fully adapt to the actual convergence search process and does not effectively coordinate global and local search. To address this issue, many researchers have proposed nonlinear improvements to the mathematical expression of *a* to enhance the algorithm’s convergence accuracy [33]. Nonlinearization of the convergence factor *a* is a common approach used to solve this equilibrium problem. The improved expression for the convergence factor *a* is as follows:

(17) $$a=2-2\left[\frac{1}{e-1}\times \left(e^{\frac{t}{T}}-1\right)\right]$$

where *a* is the convergence factor of the gray wolf algorithm; *e* is the natural constant; *t* is the number of iterations; and *T* is the maximum number of iterations.

The improved convergence method involves non-linearly decreasing the convergence factor *a* from 2 to 0 during the iteration process. This approach aligns more closely with the hunting behavior of gray wolves, where the iteration speed starts slow in the early stage and becomes faster in the later stage. This strategy enhances both the global and local search capabilities of the algorithm. By accelerating the convergence of the algorithm, it ensures better coordination between local and global search efforts.

#### 2.3.3. Improvement of Gray Wolf Iteration Weights

In the original GWO algorithm, the α, β, and δ wolves, selected based on their fitness function values, represent the current three optimal solutions. However, it is not reasonable to have these three wolves guide the other groups with the same strength during the optimization process. Such an approach would slow down the convergence of the algorithm, make it more prone to reaching local optima, and ultimately fail to produce the desired optimal solution. To address this limitation, an improved approach based on the concept of dynamic proportional weights is proposed [34]. In each iteration, the positions of the remaining wolves are dynamically guided by the distances between the three types of leading wolves and the prey. This dynamic guidance significantly enhances the algorithm’s adaptability to the environment [35]. The ω wolves update their positions as follows, under the guidance of the α, β, and δ wolves, respectively:

(18) $$X_{1}=X_{\alpha }-A_{1}D_{\alpha }$$

(19) $$X_{2}=X_{\beta }-A_{2}D_{\beta }$$

(20) $$X_{3}=X_{\delta }-A_{3}D_{\delta }$$

Based on $X_{1}$, $X_{2}$, and $X_{3}$ generated by the algorithm in each iteration, the specific formula for updating the location of ω wolf based on dynamic weights is as follows:

(21) $$\omega_{1}=\frac{\left|X_{1}\right|}{\left|X_{1}\right|+\left|X_{2}\right|+\left|X_{3}\right|}$$

(22) $$\omega_{2}=\frac{\left|X_{1}\right|}{\left|X_{1}\right|+\left|X_{2}\right|+\left|X_{3}\right|}$$

(23) $$\omega_{3}=\frac{\left|X_{1}\right|}{\left|X_{1}\right|+\left|X_{2}\right|+\left|X_{3}\right|}$$

In Equations (21)–(23), $\omega_{1}$, $\omega_{2}$, and $\omega_{3}$ correspond to the learning weights of ω wolves to α wolves, β wolves, and δ wolves, respectively. Therefore, the ω wolves finally update their positions in the following way:

(24) $$X\left(t+1\right)=\omega_{1}X_{1}+\omega_{2}X_{2}+\omega_{3}X_{3}$$

From the weight Equations (21)–(23), we can see that $\omega_{1}$, $\omega_{2}$, and $\omega_{3}$ are constantly changing during each iteration, which means “dynamic weights”. Therefore, the dynamic weights in this paper can be adjusted according to the actual convergence in each iteration so that they can be better adapted to the environment.

### 2.4. Improve GWO Algorithm to Optimize ELM

**Figure 5:**
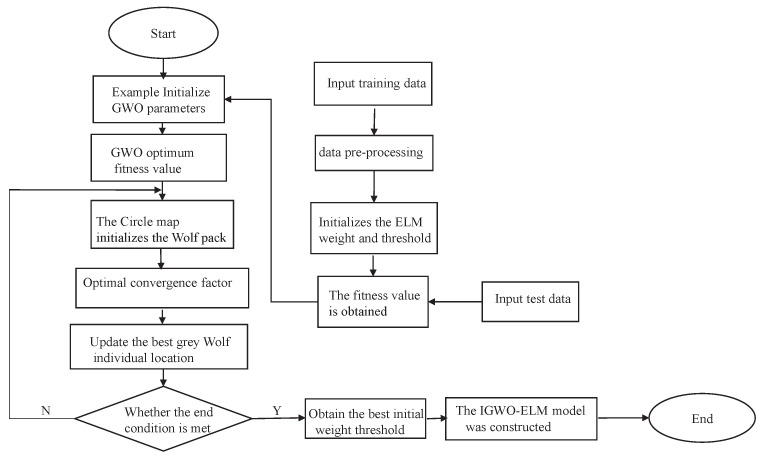
IGWO-ELM algorithm flow chart.

The improved GWO algorithm is employed to optimize the weights and thresholds of ELM, aiming to find the optimal network parameters. The weights of the input layer and the thresholds of the hidden layer serve as the location parameters for the gray wolf individuals. By applying the improved GWO algorithm, the optimal initial weights and thresholds are obtained, which are then used for training the network. This optimization process effectively mitigates the influence of randomly generated weights and thresholds on the network performance. Furthermore, GWO evaluates the fitness value based on the network error during each iteration, thereby enhancing the accuracy and stability of the model’s predictions. The fitness function is defined as follows:

(25) $$fit=\frac{1}{n}\sum_{i=1}^{n}\left|y_{i}-\hat{y_{l}}\right|$$

where $y_{i}$ is the true value, $\hat{y_{l}}$ is the predicted value, and *n* is the number of samples. The IGWO-ELM algorithm flow is shown in Figure 5 and is as follows:

1. Determining the network model structure and encoding the network weights *w* and threshold *B* to generate the gray wolf’s position vector.
2. Defining the dimensions of the weight variables dim, the population size *N*, the maximum number of iterations, and the upper and lower bounds of the search space $u_{b}$ and $l_{b}$.
3. Initialization of gray wolf populations using circle mapping to increase the population diversity of the gray wolf algorithm.
4. By optimizing the convergence factor and dynamically adjusting the gray wolf iteration weights, the optimal solution of fitness is searched in the solution space, and the location of the individual gray wolf with the optimal fitness value is used as the network initialization parameter.
5. By determining whether the iteration termination condition holds, if it does, the iteration is terminated; otherwise, the execution continues.
6. The parameters corresponding to the optimal gray wolf individuals are used as the optimal initial weights and thresholds of ELM to construct the IGWO-ELM network model.

## 3. Experiment

**Figure 6:**
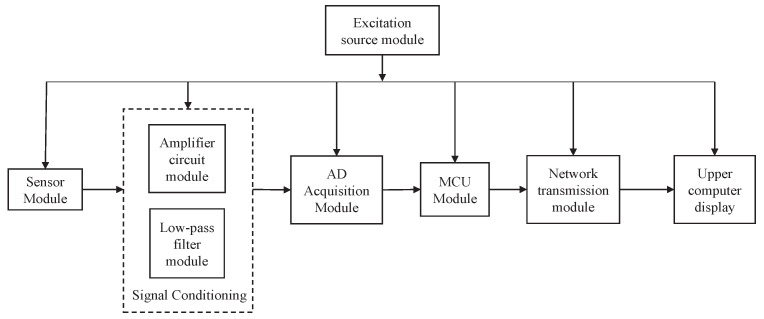
Wind tunnel balance signal detection system structure diagram.

The wind tunnel balance signal detection system comprises a six-axis sensor and a balance signal detection system board. In this study, we utilized the six-axis force/torque sensor developed by the Institute of Intelligent Mechanics (IIM) of the Chinese Academy of Sciences specifically designed for space robotic arm applications, along with a custom-designed signal detection system board. The sensor parameters are presented in Table 1 below. The balance signal detection system board consists of a signal conditioning module, excitation source module, AD acquisition module, and signal transmission module, with Ethernet transmission employed for signal transmission. The overall structure of the wind tunnel balance signal detection system is depicted in Figure 6 below.

### 3.1. Calibration and Decoupling Experiments

Calibration involves the adjustment of the equipment under test using standard metrological equipment to ensure accurate and reliable measurements. In our study, data are collected using a six-axis force/torque transducer, and precise calibration of this transducer is crucial for achieving accurate measurement performance. By calibrating the six-axis force/torque transducer, we establish the relationship between the applied force/torque and the corresponding output voltage for different force states of the transducer. This relationship can be expressed mathematically as follows:

(26) F=W·U+B

where the *W* is the weight matrix, also known as the decoupling matrix, and the *B* is the deviation vector.

A known standard force source is employed to apply a standard force/torque to the sensor in various directions, with the applied force/torque continuously varying from the minimum to the maximum measurement range. The output voltage of the sensor is simultaneously measured and recorded at each loading point. This process is repeated three times to obtain comprehensive calibration data. The calibration ranges for the platform are as follows: $F_{x}/F_{y}$—1000 N; $F_{z}$—2000 N; and $M_{x}/M_{y}/M_{z}$—50 Nm. There is an accuracy of 0.1${\%}_{FS}$.

After the calibration experiments are completed, the decoupling matrix *W* deviation vector *B* is calculated using a linear neural network as follows:

(27) $$W=\left\{\begin{array}{cccccc} -12.833 & 0.115 & -0.113 & 0.122 & -17.068 & -0.130\\ 0.093 & -12.90 & -0.515 & 16.741 & 0.208 & 0.034\\ -0.076 & -0.501 & -9.964 & 0.122 & 0.023 & 0.001\\ 0.002 & -0.028 & -0.017 & 0.128 & 0.004 & -0.001\\ 0.026 & 0.002 & -0.001 & -0.003 & 0.132 & 0.001\\ 0.037 & -0.001 & -0.003 & 0.001 & 0.002 & -0.123 \end{array}\right\}$$

(28) $$B=\left[38203.083,-4828.718,13017.101,-112.087,-201.395,-162.169\right]$$

Finally, the transfer expression (26) is obtained.

### 3.2. High-Low Temperature Experiment

To investigate the temperature characteristics of the wind tunnel balance signal detection system, the drift resulting from system measurement errors is analyzed through high-low temperature experiment. The obtained data are then used to train a model. In order to accurately study the temperature-induced drift characteristics, no force/torque is applied to the six-axis force/torque sensor, allowing the system output to vary solely due to temperature changes.

**Figure 7:**
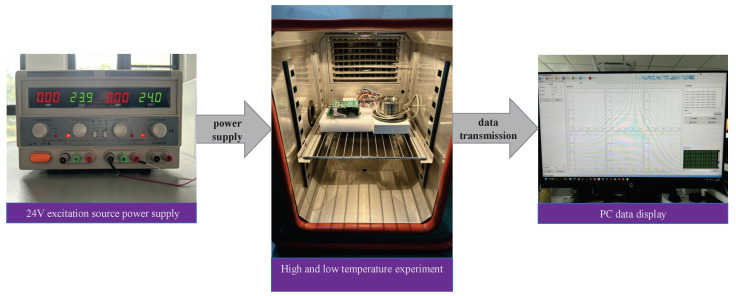
High–low temperature experiment demonstration.

The wind tunnel balance signal detection system is placed inside a sealed high-low temperature experimental chamber, with the system powered by a 24 V DC power supply. The temperature is varied in increments of 20 °C, ranging from −40 to 80 °C. To ensure accurate temperature measurements, each sampling point is maintained for 2 h. The sampling points include −40, −20, 0, 20, 25, 40, 60, and 80 °C. The acquisition module transmits the voltage output values at each temperature to the computer via the network for storage and processing. The high–low temperature experimental setup is depicted in Figure 7.

In this paper, the sensor measurement error $E_{m}$ is employed as the evaluation index for quantifying the degree of temperature-induced drift in each bridge of the wind tunnel balance signal detection system. The defined equation is as follows:

(29) $$E_{m}=\frac{U_{T}-U_{ref}}{U\left(FS\right)}\times 100\%$$

where $U_{T}$ is the measured value of the wind tunnel balance signal detection system at T°C; $U_{ref}$ indicates the measured value at the standard temperature, i.e., 25 °C; and $U\left(FS\right)$ indicates the range of the wind tunnel balance signal detection system.

**Figure 8:**
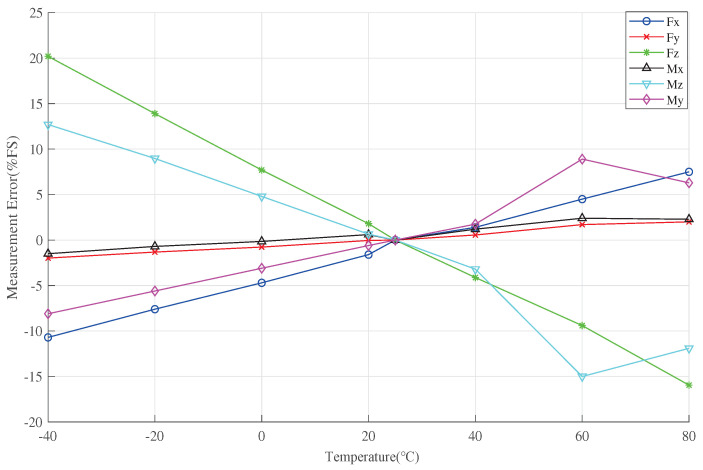
Uncompensated measurement error.

The temperature-induced drift caused by measurement errors in the six dimensions can be observed using Equation (27) as depicted in Figure 8. From the figure, it is evident that the temperature-related errors exhibit predominantly nonlinear behavior. With increasing the temperature, the errors of $F_{x}$ and $M_{z}$ gradually transition from negative to zero and then increase; the errors of $F_{z}$ and $M_{y}$ follow a similar trend, but with opposite correlation; the errors of $F_{y}$ and $M_{X}$ remain relatively smaller as the temperature increases. Among these, $F_{z}$ exhibits the largest error, reaching up to 20${\%}_{FS}$, while $F_{x}$ and $M_{y}$ also have maximum errors of 10${\%}_{FS}$. In short, the temperature-induced error can reach a minimum of $0.6{\%}_{FS}$. Furthermore, temperature introduces significant nonlinearity-induced errors, significantly impacting the accuracy of the wind tunnel balance signal detection system.

## 4. Temperature Compensation

### 4.1. Wind Tunnel Balance Signal Detection System Compensation Principle

**Figure 9:**
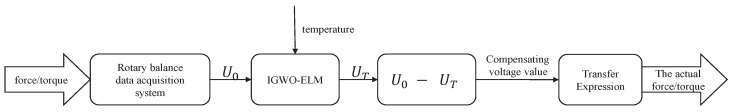
IGWO-ELM temperature compensation system diagram.

To mitigate the impact of temperature-induced drift, temperature compensation is necessary for the wind tunnel balance signal detection system. The temperature compensation process is illustrated in Figure 9. Initially, the force and torque measured by the system are converted to a voltage output $U_{0}$. Subsequently, the voltage output and temperature are compensated using a model to obtain the thermal output $U_{T}$. Finally, the measured output is subtracted from the thermal output to obtain the actual output. The compensated output is determined using Equation (26).

### 4.2. Compensation Model

Firstly, the acquired data are divided into training and test sets using the leave-out method. In this process, 80% of the acquired data is utilized to train the IGWO-ELM algorithm, while the remaining 20% is reserved to assess the accuracy of the compensation. Both the training and test sets consist of measured output and temperature values for each direction. Furthermore, to validate the prediction performance of the IGWO-ELM algorithm, BPNN, GWO-ELM, PSO-ELM, and GWO-RBFNN are trained using the same data samples, respectively.

The parameters of the GWO algorithm are set as follows: the number of populations is 30, the upper limit is 1, the lower limit is 0, and the number of iterations is 200. The parameters of the PSO algorithm are set as follows: $c_{1}$ = $c_{2}$ = 1.49445, the number of populations is 30, the maximum value of individuals popmax = 1, the minimum value of individuals popmin = 0, the maximum value of individuals velocity $V_{max}$ = 100, the minimum value $V_{min}$ = −100, and the maximum number of iterations maxgen = 200. The implicit layer neurons of ELM are set to 8, the implicit layer activation function is sigmoid, and the RBFNN centroids are −40, −20, 0, 20, 25, 40, 60, and 80. All parameters are kept the same as much as possible.

In addition, the performance of the algorithm is evaluated by means of the mean square error (MSE) as a fitness function, which is calculated as follows:

(30) $$MSE=\frac{1}{n}\sum_{i=1}^{n}{\left(y_{i}-\hat{y_{l}}\right)}^{2}$$

where $y_{i}$ is the true value of the *i*-th sample, $\hat{y_{l}}$ is the predicted value of the *i*-th sample, and *n* is the number of samples.

### 4.3. Compensation Results and Analysis

The compensation results for each dimension in the test sets are presented in Table 2 below. Among them, the test results of IGWO-ELM demonstrate the best performance, while ELM and GWO-RBFNN perform relatively worse. From the test results, it can be observed that the errors of IGWO-ELM in the four dimensions of $F_{x}$, $F_{y}$, $M_{y}$, and $M_{z}$ can be limited to within 0.01${\%}_{FS}$. Furthermore, the test results of IGWO-ELM outperform those of single GWO-ELM, showcasing effective compensation for temperature-induced errors. It is evident that under the same network structure conditions, the performance of GWO-ELM is not as satisfactory, while the performance of GWO-ELM is superior to that of GWO-RBFNN.

By examining the data presented in Table 2, it becomes evident that the proposed IGWO-ELM algorithm in this study exhibits superior performance in compensating for temperature variations as reflected by the smaller error indicators compared to other algorithms. Of particular importance is the significant reduction in compensation errors for $F_{x}$, $F_{y}$, $M_{y}$, and $M_{z}$, which are decreased by 20% when compared to the original standard ELM algorithm. This signifies the effectiveness of the proposed IGWO-ELM algorithm in optimizing temperature compensation for the wind tunnel balance data acquisition system. As a result, the algorithm utilizing IGWO-ELM outperforms GWO-ELM, PSO-ELM, and GWO-RBFNN in achieving accurate temperature compensation.

**Figure 10:**
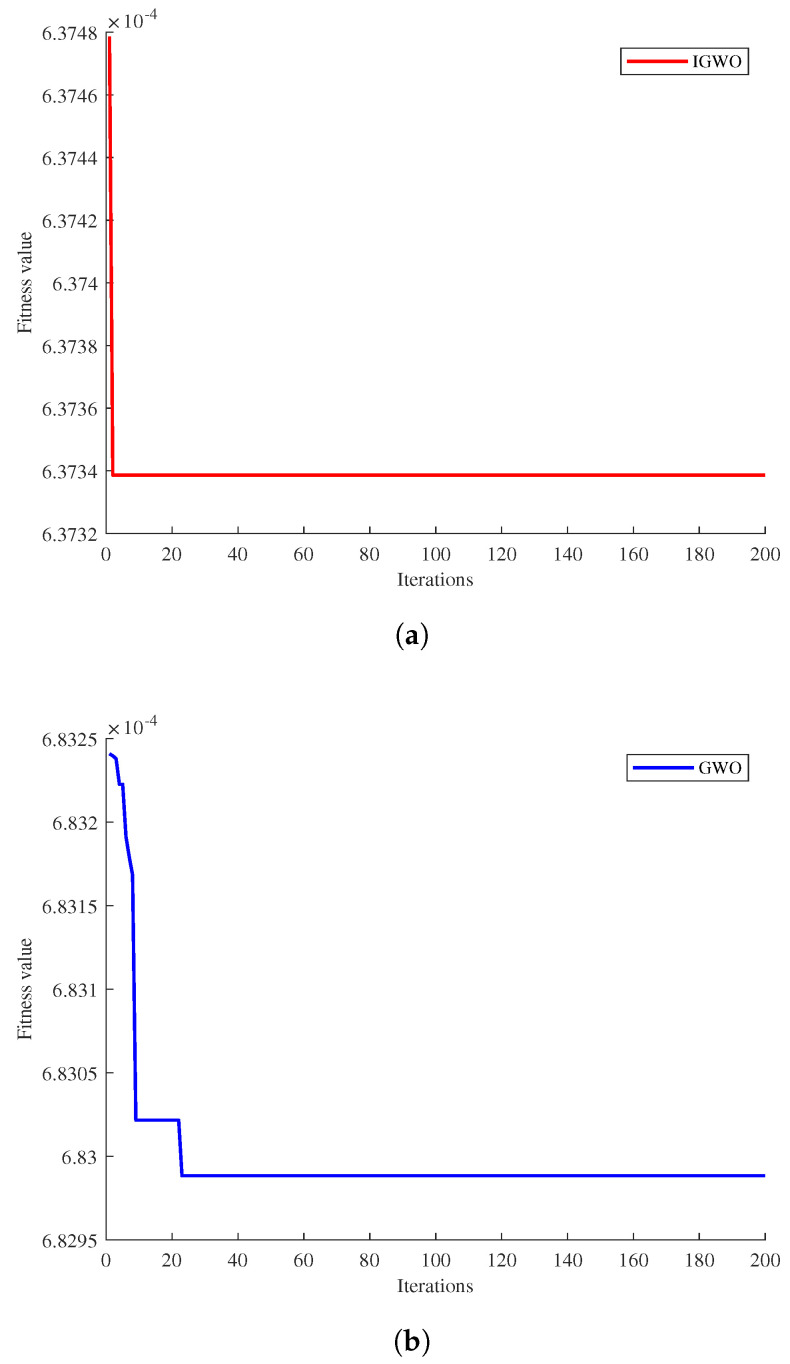
Adaptability curve. (**a**) Improved GWO adaptation curve. (**b**) GWO adaptation curve.

**Figure 11:**
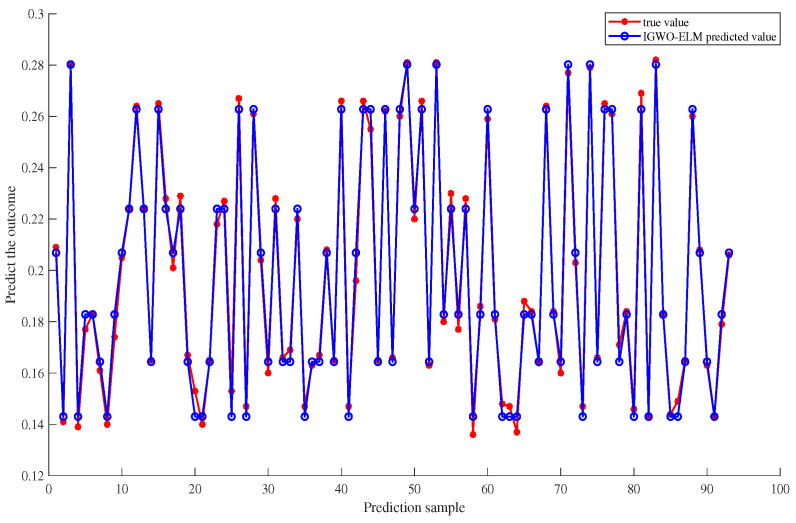
Comparison of predicted values and actual graphs.

From the data presented in the table above, it is evident that the iterations in the $F_{y}$ dimension yield the best results. Therefore, $F_{y}$ is chosen as an example to evaluate the convergence and ability to escape local optima of the IGWO algorithm after compensation. The iterative convergence curve of the algorithm is depicted in Figure 10, where the horizontal axis represents the number of iterations, and the vertical axis represents the fitness value. From the figure, it can be observed that the IGWO algorithm shows faster convergence and a smooth curve in the temperature compensation model. A lower fitness value indicates higher optimization search accuracy. The appearance of the curve’s inflection point signifies faster convergence speed. The smooth curve suggests that the algorithm excels in escaping local optima. After conducting several experiments, a comparison between the predicted and actual values of the test samples can be obtained as shown in Figure 11.

**Figure 12:**
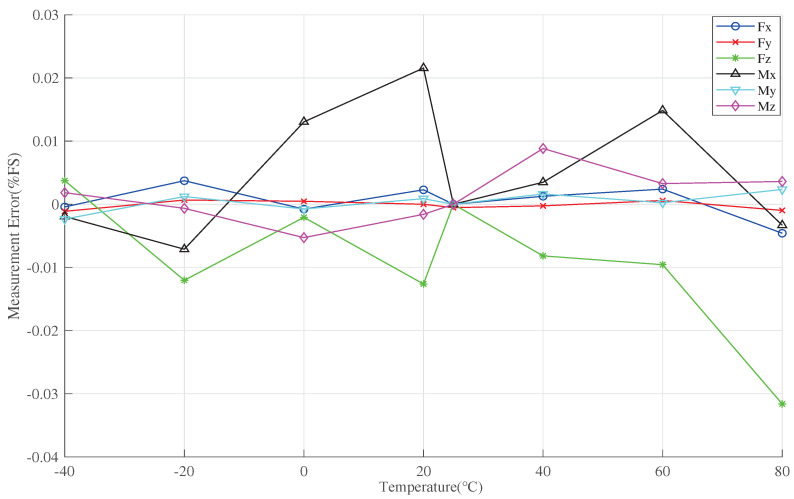
Measurement error after compensation by IGWO-ELM algorithm.

The measurement errors of the wind tunnel balance signal detection system after compensation using IGWO-ELM are illustrated in Figure 12. Overall, the measurement errors in all dimensions are significantly reduced after the application of IGWO-ELM compensation. The best compensation effect is achieved in the $F_{y}$ dimension, with a value of $2.5\times 10^{-3}{\%}_{FS}$. The errors in all other dimensions are within $0.04{\%}_{FS}$. As a result, the measurement data obtained after the implementation of the IGWO-ELM compensation algorithm are largely independent of the temperature, reducing the measurement error from 20${\%}_{FS}$ to within 0.04${\%}_{FS}$, effectively controlling the measurement error caused by temperature.

## 5. Conclusions

In this paper, aiming at the temperature error problem of the wind tunnel balance signal detection system, a temperature compensation method is proposed. The method is based on an optimized extreme learning machine using an IGWO algorithm. The research focuses on a wind tunnel balance signal detection system that consists of a six-axis force/torque sensor applied to a space robotic arm and a signal detection system board. High–low temperature experiments are conducted to collect experimental data. For temperature compensation, various algorithms, including ELM, GWO-ELM, PSO-ELM, GWO-RBFNN, and IGWO-ELM, are employed and compared. The experimental results demonstrate that IGWO-ELM outperforms other algorithms in terms of prediction ability and reduces the temperature-induced error to within 0.04${\%}_{FS}$. By improving the initialization of the gray wolf population, optimizing the convergence factor, and adapting iteration weights, the IGWO-ELM algorithm exhibits improved stability, is less likely to converge to local optimal solutions, and determines the optimal network parameters for the ELM. In short, the experimental results confirm that the proposed IGWO-ELM temperature compensation algorithm effectively addresses the temperature error issue and provides better temperature compensation performance with enhanced stability.

Although the temperature compensation achieved by the IGWO-ELM algorithm is superior, its controllable performance is relatively weak, and its ability to adapt to different characteristics of the dataset is not highly effective. Future research can focus on enhancing the controllability of the IGWO-ELM algorithm, particularly in relation to its adjustment based on the specific characteristics of the dataset. This investigation will contribute to further improving the performance and versatility of the IGWO-ELM algorithm in temperature compensation applications.

## Figures and Tables

**Table 1:** Six-axis force/torque sensor parameters.

Measurement Range $\left(\pm \right)$	$F_{x}/F_{y}/F_{z}$: 1000 N, $M_{x}/M_{y}/M_{z}$: 40 Nm
Dimensional Parameters	Height: 53 mm, Diameter: 93 mm
Overload capacity	≤$300{\%}_{FS}$
Zero output	≤$\pm 0.5{\%}_{FS}$
Supply voltage/power	DC 5V/2W

**Table 2:** Comparison of prediction errors of different algorithms.

Parameters	ELM	GWO-ELM	IGWO-ELM	PSO-ELM	GWO-RBFNN
$F_{x}$	$3.7567\times 10^{-5}$	$3.3718\times 10^{-5}$	$3.0089\times 10^{-5}$	$3.5404\times 10^{-5}$	$3.7606\times 10^{-5}$
$F_{y}$	$1.8891\times 10^{-5}$	$1.7274\times 10^{-5}$	$1.3948\times 10^{-5}$	$1.7350\times 10^{-5}$	$1.6507\times 10^{-5}$
$F_{z}$	$3.5412\times 10^{-4}$	$3.4942\times 10^{-4}$	$2.8792\times 10^{-4}$	$3.2449\times 10^{-4}$	$3.2074\times 10^{-4}$
$M_{x}$	$2.5310\times 10^{-4}$	$2.5227\times 10^{-4}$	$1.9278\times 10^{-4}$	$2.4042\times 10^{-4}$	$2.2995\times 10^{-4}$
$M_{y}$	$2.2462\times 10^{-5}$	$1.9885\times 10^{-5}$	$1.7164\times 10^{-5}$	$1.9339\times 10^{-5}$	$1.9990\times 10^{-5}$
$M_{z}$	$7.2498\times 10^{-5}$	$6.8145\times 10^{-5}$	$6.2343\times 10^{-5}$	$6.7122\times 10^{-5}$	$7.2946\times 10^{-5}$

## Data Availability

Not applicable.
